# Cladistics of the *Zethus spinosus* de Saussure Species Group: A New Subgenus of *Zethus* Fabricius, 1804 (Hymenoptera, Vespidae)

**DOI:** 10.3390/insects17090898

**Published:** 2026-08-27

**Authors:** Ana Helena Cruciol, Fernando Barbosa Noll, Rogério Botion Lopes

**Affiliations:** 1 Departamento de Ciências Biológicas, Instituto de Biociências, Letras e Ciências Exatas, Universidade Estadual Paulista “Júlio de Mesquita Filho”, São José do Rio Preto 15054-000, Brazil; fernando.noll@unesp.br; 2 Campus de Arapiraca, Universidade Federal de Alagoas, Arapiraca 57309-005, Brazil; rogerio.lopes@arapiraca.ufal.br

**Keywords:** phylogeny, zethini, morphological analysis

## Abstract

*Zethus* Fabricius is the largest genus of vespid wasps with over 300 species worldwide that are divided into subgenera and further into species groups. Recent phylogenetic studies raised the number of subgenera from four to nine, six of which are in the Neotropics. However, three Neotropical species groups were not assigned to any subgenus due to lack of node support. The *Zethus spinosus* species group is such a case and, while previously inserted in the nominotypical subgenus, it was recovered closer to *Zethus* (*Zethusculus*), but with no support. Thus, a phylogenetic parsimony analysis of morphological data of the group was carried out with better ingroup and outgroup sampling for testing to find a more robust resolution for the group’s placement. We recovered the *Zethus spinosus* group as monophyletic and as a sister group of *Zethus* (*Zethusculus*). In both cases, it was not only unique synapomorphies that supported the clades, but symmetric resampling also recovered significantly better values. Therefore, to maintain all taxa monophyletic, a new subgenus, “*Zethus* (*Zethittus*)” Cruciol & Lopes, subg. n., is described to accommodate the *Zethus spinosus* group. Additionally, a new species, *Zethus* (*Zethittus*) *ramphoceros* Cruciol & Lopes, sp. nov. from Brazil is described herein. The present study also provides a new record of *Zethus semiplanus* Cruciol et al. (2026).

## Simple Summary

This paper focuses on a phylogenetic analysis of the *Zethus spinosus* species group (Hymenoptera, Vespidae), including the proposal of a new subgenus. *Zethus* is the largest genus of Eumeninae wasps in the Neotropical region, comprising approximately 300 species worldwide. This study investigated the evolutionary relationships within the *Zethus spinosus* group and contributed to a better understanding of its diversity and classification in the taxonomy of aculeate wasps.

## 1. Introduction

The Zethines include the most diverse genus of the subfamily, *Zethus* Fabricius, 1804 and the distribution of *Zethus* extends across much of the world, with particularly high species diversity in the Neotropical region [1,2]. De Saussure [3] began the process of subdividing *Zethus* and proposed internal divisions that were later reorganized into five groups [4]. Despite subsequent modifications, these divisions did not correspond to the subgenera currently recognized; among them, only *Zethusculus* was later elevated to subgeneric status, although with a composition different from that originally proposed [5].

Bohart & Stange [6] revised *Zethus* in the New World, organizing the species into 29 species groups and establishing three subgenera: *Zethus*, *Zethusculus* and *Zethoides*. Later, Giordani Soika [7] described *Madecazethus*, a subgenus restricted to Madagascar. Afterward, Lopes e Noll [5] synthesized the incongruences in the current classification and focused on relationships in the subgenus *Zethus* (*Zethoides*). Following that, Lopes et al. [8] conducted the first phylogenetic analysis of the genus *Zethus* Fabricius, 1804. Although the monophyly of *Zethus* was not initially recovered, taxonomic adjustments, including changes in subgeneric assignments and the synonymization of *Ischocoelia* and *Ctenochilus,* restored the taxon as a monophyletic group. Based on these results, Lopes et al. [7] proposed a new classification for the genus *Zethus*, recognizing six Neotropical subgenera: *Zethus* Fabricius, 1804; *Zethusculus* de Saussure, 1855; *Zethoides* Fox, 1899; *Ctenochilus* de Saussure, 1856; *Didymogastra* Perty, 1833; and *Wettsteinia* Dalla Torre, 1904. 

Despite these advances, three Neotropical species groups could not be assigned to any subgenus because their phylogenetic positions lacked statistical support. The *Zethus spinosus* group is one such example: its recovered position in the phylogeny suggests a closer relationship with *Zethusculus,* but it received low phylogenetic support, according to Lopes et al. [8]. The *Zethus spinosus* group is distributed across tropical regions of North and South America [6]. Recently, the taxonomic knowledge of the group was improved by the description of five new species by Cruciol et al. [9]. Nevertheless, more novelties awaited as one more new species was found. 

Expanding on previous work on the study of the group, we provide additions to its taxonomy and propose a phylogenetic hypothesis for this group. The present paper aimed to test its monophyly and to investigate the phylogenetic relationships among species and other subgenera. Herein, we present the phylogeny along with the descriptions of a new subgenus *Zethus* (*Zethittus*) subg. nov. Additionally, another new species is described, a new record of *Zethus semiplanus* Cruciol & Lopes, 2026. 

## 2. Materials and Methods

### 2.1. Taxon Sampling

A total of 62 specimens fitting the *Zethus spinosus* group were examined. The list of examined material corresponds to those specimens reported by Cruciol et al. [9] with the inclusion of the new species, from the following institutions:

BME—Bohart Museum of Entomology, University of California, Davis, CA, USA.
DZSJRP—Coleção de Hymenoptera de São José do Rio Preto, Instituto de Biociências, Letras e Ciências Exatas, Universidade Estadual Paulista “Júlio de Mesquita Filho”, São José do Rio Preto, Brazil.
DZUP—Coleção Entomológica Padre Jesus Santiago Moure, Universidade Federal do Paraná, Brazil.
FSCA—Florida State Collection of Arthropods, Gainesville, FL, USA.
IBNPY—Museo Nacional de Historia Natural del Paraguay, San Lorenzo, Paraguay.
INPA—Instituto Nacional de Pesquisa da Amazônia, Manaus, Brazil.
MPEG—Museu Paranaense Emílio Goeldi, Belém, Pará.
MSNG—Museo Civico di Storia Natureale “Giacomo Doria”, Genova, Italy.
RPSP—Coleção Entomológica Prof. J.M.F. Camargo, Faculdade de Filosofia, Ciências e Letras de Ribeirão Preto, Universidade de São Paulo, Ribeirão Preto, Brazil.

For comparison, the holotype of *Zethus coroicae*, Bohart & Stange, 1965, deposited in the Entomological Collection of The Swiss Federal Institute of Technology (Zurich, Switzerland) was requested. However, only a few photographs of the specimen were obtained which were insufficient and unclear to justify inclusion in the analyses. 

Additionally, 16 terminals were used as outgroups, including the following: *Z.* (*Ischnocoelia*) *fulvus* von Schultess, 1910; *Z.* (*Ctenochilus*) *pilipalpus* Spinola, 1851; *Z.* (*Zethus*) *cylindricus* Fox, 1899; *Z.* (*Zethoides*) *diminutus* Fox, 1899; *Z.* (*Zethoides*) *aztecus* Saussure, 1857; *Z.* (*Zethus*) *fuscus* Perty, 1833; *Z.* (*Zethus*) *smithii* Saussure, 1855; *Z.* (*Zethusculus*) *imperfectus* Fox, 1899; *Z.* (*Zethusculus*) *mexicanus* Linnaeus, 1767; *Z.* (*Zethusculus*) *romandinus* Saussure, 1852; *Z.* (*Zethus*) *spinipes* Say, 1837; *Z.* (*Zethus*) *infundibuliformis* Fabricius, 1804; *Z.* (*Zethus*) *coeruleopennis* Fabricius, 1798; *Z.* (*Zethus*) *prominens* Fox, 1899; *Z.* (*Zethus*) *discoelioides* Saussure, 1852 and *Z.* (*Zethus*) *wagneri* Bohart & Stange, 1965. Several taxons were used as outgroups due to the low phylogenetics support of *Zethus spinosus*. 

*Zethus* (*Ischnocoelia*) *fulvus* was used to root the tree because it is regarded as the sister group of the Neotropical clade by Lopes et al. [8].

### 2.2. Character Circumscription

The analysis was based on morphological characters from both external morphology and male genitalia. Male genitalia were treated in KOH 10% for 24 h, followed by neutralization with Acetic Acid 10% solution, rinsed in water and then stored in glycerin. After this, the genitalia were examined using a stereoscope Leica M205C (Wetzlar, Germany) and photographs (digital camera Leica FlexCAMC1). Images were captured with the Leica Application Suite-X (LAS) software and processed using the Adobe Photoshop 2026 version 27.9.1. When necessary and possible, drawings were made from photographs to facilitate the understanding of the morphology.

Terminology follows the basic literature, Bohart & Stange [6], Carpenter & Marques [1], Picket & Carpenter [10], Hermes et al. [11], Lopes & Noll ([5], Lopes et al. [7], Lopes et al [12], Cruciol et al. [9] and, for the genitals, follows Bitsch [13]. However, adjustments were made to the new characteristics of the species in the group. Abbreviations are tergite (T) and sternite (S), followed by their respective ordinal numbers.

### 2.3. Phylogenetic Analysis

Following circumscription, characters were coded and inserted into a matrix using the Winclada software (Version 1.00.08) [14] and exported for Tree Analysis using New Technology (TNT) [15]. An implied weighting [16] -scheme was carried out in order to down-weight homoplastic characters using the script “setk.run” by Salvador Arias, based on Goloboff et al. [17]. New technology search algorithms were used in default settings, as per the except specified below: Ratchet with 200 iterations with up–down perturbation 8; drift with 20 cycles. Tree fusing with 10 rounds; Maximum trees held in memory 10,000, random seed 0 (zero). When more than one tree with the minimum number of steps was recovered, a strict consensus tree was obtained using the options Trees/ Consensus/ Calculate = Strict Using = all, Include = all taxa. Support values were estimated through symmetric resampling (Goloboff) [18] with 10,000 replications and the values of contradictory groups (CG) were presented for clade support.

After the search was completed, the resulting tree was opened in Winclada and exported for editing in Adobe Illustrator, the 2026 30.7 version.

## 3. Results

### 3.1. Phylogenetic Analysis

**Figure 1:**
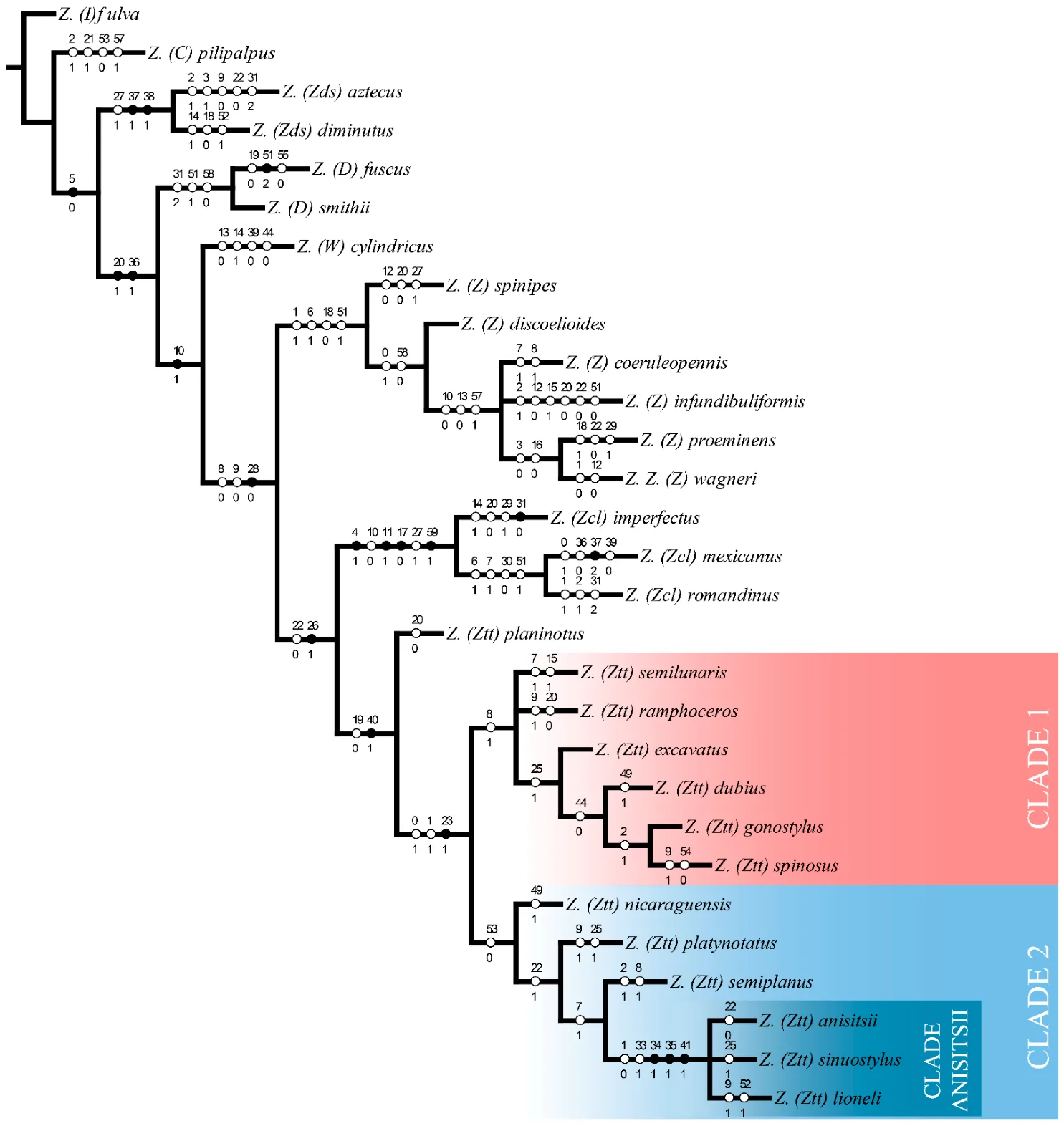
Consensus of the six most parsimonious trees under implied weighting with k = 3.75. Subgenera are abbreviated: C—*Ctenochilus*, D—*Didymogastra*, I—*Ischonocoleia*, W—*Wettsteinia*, Z—*Zethus*, Zcl—*Zethusculus*, Zds—*Zethoides*, Ztt—*Zethittus*. White circles are homoplasies while black are unique synapomorphies. Numbers above circles indicate character number and below are character states. The empty circles represent homoplastic synapomorphies, while the filled circles indicate unique synapomorphies.

A total of 60 characters were circumscribed, 15 being from the head, 16 from the mesosoma, 13 from the metasoma and 16 from the male genitalia (the character list is provided in Appendix A). The matrix is provided in the Supplementary Materials in “tnt” format. The characters were based on the studies and terminology described in the Methodology Section 2.2, particularly those presented by Bohart and Stange [6], Lopes et al. [8] and Cruciol et al. [9]. The script suggested a k = 3.75 for which six trees were recovered and whose consensus is presented in Figure 1.

**Figure 2:**
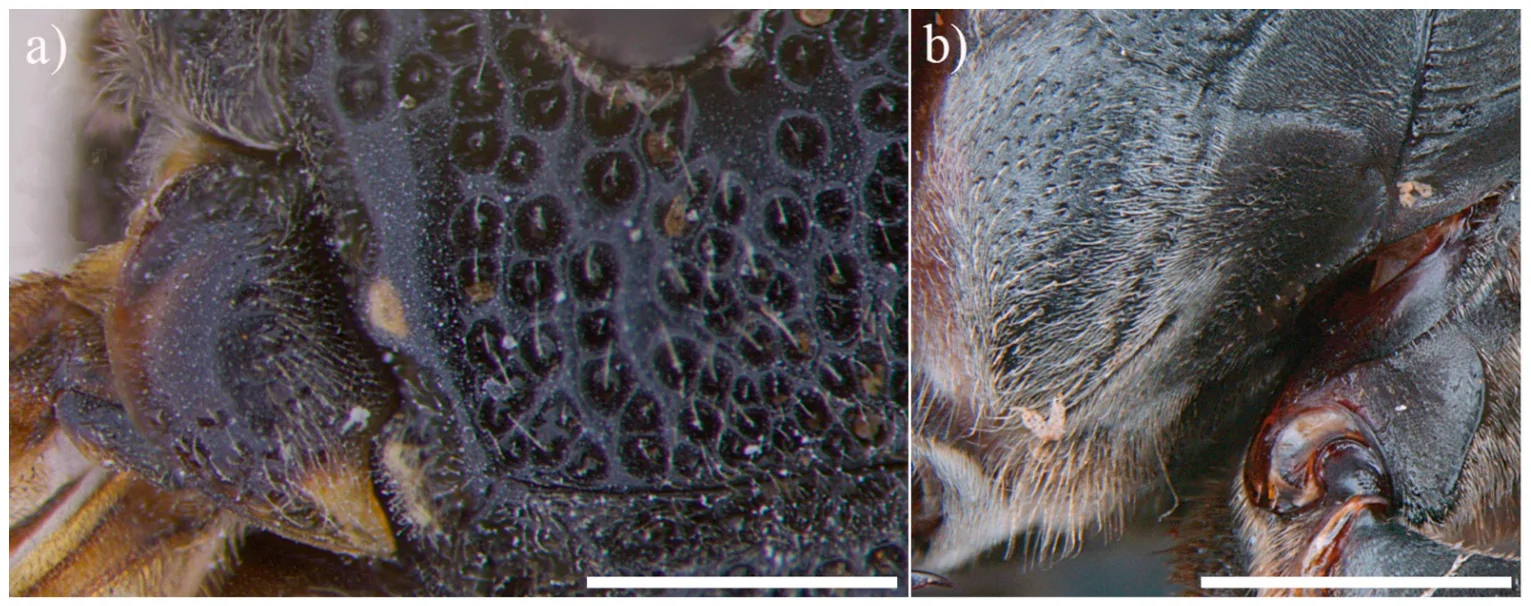
Synapomorphies supporting the sister-group relationship of *Z*. *spinosus* group with the subgenus *Zethusculus*. *Zethus anitisii*: (**a**) presence of only one projection, but discoidal punctation is absent; *Zethus mexicanus*: (**b**) presence of an anterior carina on the fore coxa. Scale bars: 1.0 mm.

The phylogenetic analysis recovered six equally parsimonious phylogenetic trees. The strict consensus tree confirmed the monophyly of the *Zethus spinosus* group (Figure 1)**.** This relationship is supported by the synapomorphies recovered for characters 19 and 40, corresponding to the epicnemial carinae, dorsal portion incomplete, and female T6, subapical longitudinal depression present, respectively (character list available in Appendix A). Furthermore, the analysis recovered this taxon as the sister group of the subgenus *Zethusculus*, which was also recovered as monophyletic. This relationship is supported by the synapomorphies recovered for characters 22 and 26, corresponding to mesoscutum, parapsidal lines present, and fore coxae, anterior carinae present, respectively (Figure 2). Therefore, the phylogenetic evidence obtained here supports the recognition of the *Zethus spinosus* group as a distinct and previously unrecognized subgenus within *Zethus*, which is herein established as *Zethittus*.

### 3.2. Taxonomy

As a result, this section presents the description of the new subgenus, description of a new species, and a new record of *Zethus semiplanus* Cruciol & Lopes, 2026.

 

*Zethus* (*Zethittus*) Cruciol & Lopes, subg. nov.

 

**Type-species:** *Zethus spinosus* Saussure, 1857.

 

**Figure 3:**
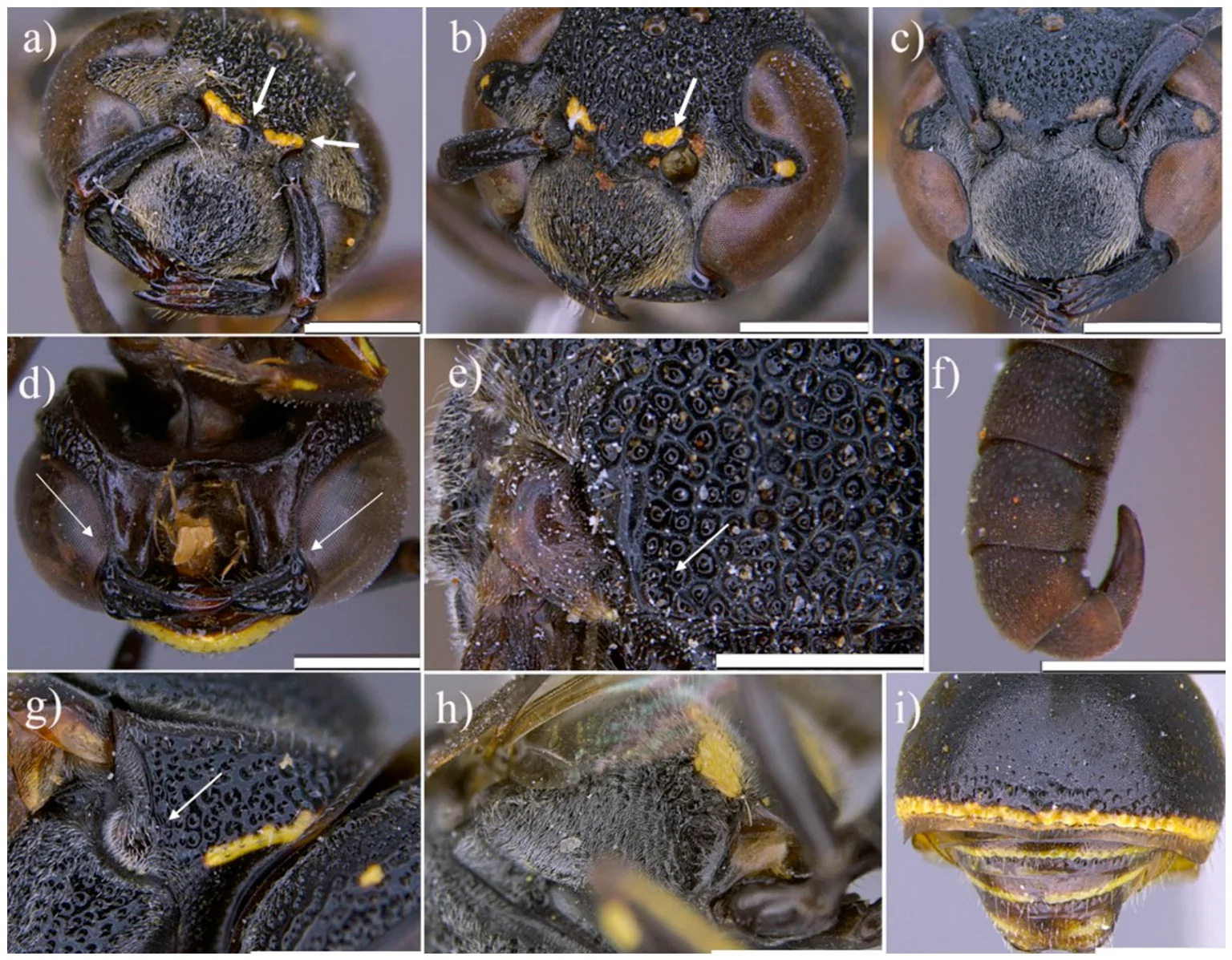
Diagnosis characters of *Zethus* (*Zethittus*). *Zethus spinosus*: (**a**) longitudinal and transversal, internatennal carinae; *Zethus excavatus*: (**b**) only longitudinal interantennal carinae; *Zethus anisitsii*: (**c**) internantennal region exhibiting just an area elevated; Zethus semilunaris: (**d**) hypostomal carinae; *Zethus semilunaris:* (**e**) parapsidal lines; *Zethus dubius*: (**f**) F-XI dorsoventrally flattened; *Zethus nicaraguenses*: (**g**) pretegular carinae; *Zethus lioneli*: (**h**) propodeum with irregular punctates; *Zethus sinuostylus*: (**i**) T2 with macropunctate band. Scale bars: 1.0 mm.

**Diagnosis:** The male S7 has a large and shallow notch and shows a translucent lamella and the overdeveloped genitalia compared to other groups, especially in gonocoxite. The development of male genitalia is unique to the taxon. The male flagellomere XI shows dorsoventral flattening (Figure 3). The female presents the clypeus-only punctate, without striae and the T6 with an obtuse longitudinal median depression (Figure 3).

**Figure 4:**
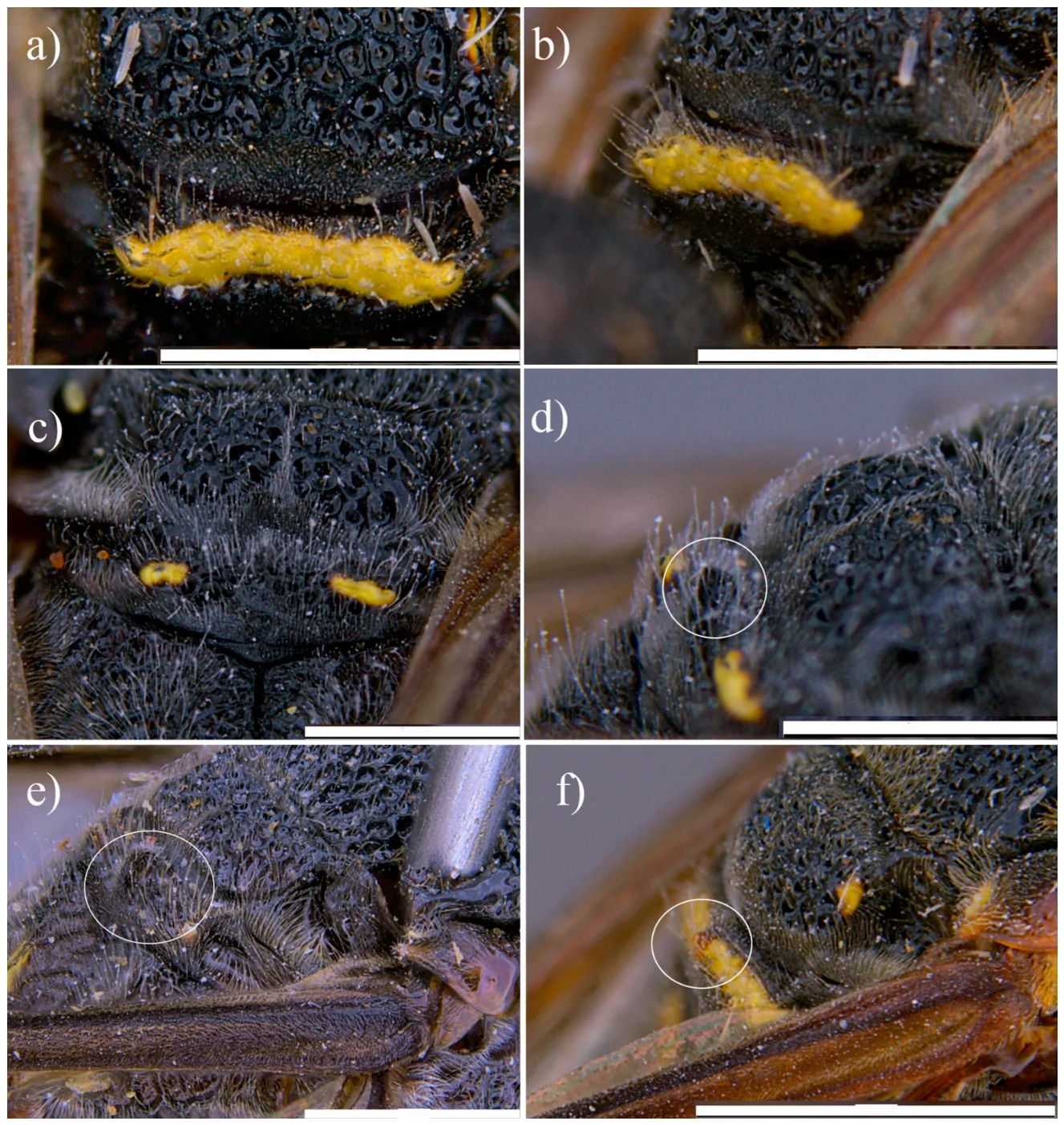
Differents tubercle shape. *Zethus planinotus*: (**a**) and (**b**) absent tubercle; *Zethus spinosus*: (**c**) proeminent tubercle; *Zethus spinosus*: (**d**) median lamelar tubercle; *Zethus seminlunaris*: (**e**) strongly and robust tubercle; *Zethus nicaraguenses*: (**f**) weakly and acute tubercle. Scale bars: 1.0 mm.

**Description:** Black body, polished with yellow spots in the clypeal region in males; spots on the metanotum; and bands on the legs, especially on the femur and apical bands on the tergites and sternites. The body is generally macropunctate, except in the female, in which clypeus is only punctate, without striae; in the mesoscutum, some “interruptions of punctation” can also be observed and T2 shows dense micropunctations; however, the apical region presents a band of macropunctation, in some species irregular or homogeneous (Figure 3). Interantennal carinae present or absent; when present, comprising both longitudinal and transverse carinae. Additionally, the region may present only an elevation where the carinae would be. Hypostomal carinae are present and complete. On the mesoscutum, notaulices are absent; most species present parapsidal lines. The female may have discal punctation; however, most exhibit a smooth area (without punctation) external to the parapsidal line, where discal punctation would be located (Figure 3). In the legs: anterior coxa with an anterior carina (Figure 2). Propodeum with an irregular lateral carina, most often with a “cranulated” appearance (Figure 3). The male presents flagellum in a hook shape, F-X symmetrical, F-XI showing dorsoventral flattening (Figure 3); tegula with the outer margin elevated only in the posterior half and pretegular carina present. The subhumeral area is reduced and delimited by a “fold” at the apex of the humerus. The metanotum presents a median tubercle (Figure 4) except *Zethus planinotus*. The tubercle may be prominent, reduced or obsolete (Figure 4); mid-tibia with two apical spurs; hind tibia with dispersed spines. Propodeum with a slight or moderate depression. The propodeal lamella is absent and the dorsal opening of the propodeum is present. The T2 stem is shorter than the T1 stem. T3 with gradulus. Female T6 has a subapical longitudinal depression. Compounding the characteristic of the group, S6 of the female has a depression that may be shallow or deep, showing lateral tubercles and the male S7 also has a notch exhibiting a translucent lamella which follows the notch.

Pilosity is present over the entire body, mostly sparse and medium-sized, being dense in the lateral region of the clypeus and in females around T6 and S6. There is an exceptional development of gonocoxite. The gonostylus presents an arched structure, which varies in thickness. The ventral lobe is present, but can be fused to the apodeme.

**Distribution:** The subgenus is represented in the tropical region of the Americas, being recorded from South America (from Argentina) to North America, in the southern region of Mexico.

**Etymology:** Due to its small size, a diminutive form reference of genus *Zethus* in Latin was chosen.

**Included species:** The subgenus is exclusively composed of species belonging to the *Zethus spinosus* group, newly described species according to Cruciol et al. (2026) [8] and the new species described include the following: *Zethus anisitsii* Brèthes, 1906; *Zethus coroicae* Bohart & Stange, 1965; *Zethus dubius* Smith, 1857; *Zethus excavatus* Bohart & Stange, 1965; *Zethus gonostylus* Bohart & Stange, 1965; *Zethus nicaraguenses* Zavattari, 1912; *Zethus spinosus* Saussure, 1857; *Zethus sinuostylus* Lopes, 2014; *Zethus lioneli* Cruciol & Lopes, 2026; *Zethus platinotatus* Cruciol, Stange & Lopes, 2026; *Zethus platynotatus* Cruciol, Stange & Lopes, 2026; *Zethus semilunaris* Cruciol, Stange & Lopes, 2026, *Zethus semiplanus* Cruciol & Lopes, 2026 and *Zethus ramphoceros* Cruciol, Noll & Lopes, 2026.

**Observations:** Since the subgenus includes a single species group, their diagnoses overlap. 

 

*Zethus (Zethittus) ramphoceros* Cruciol & Lopes, **sp. nov**.

**Figure 5:**
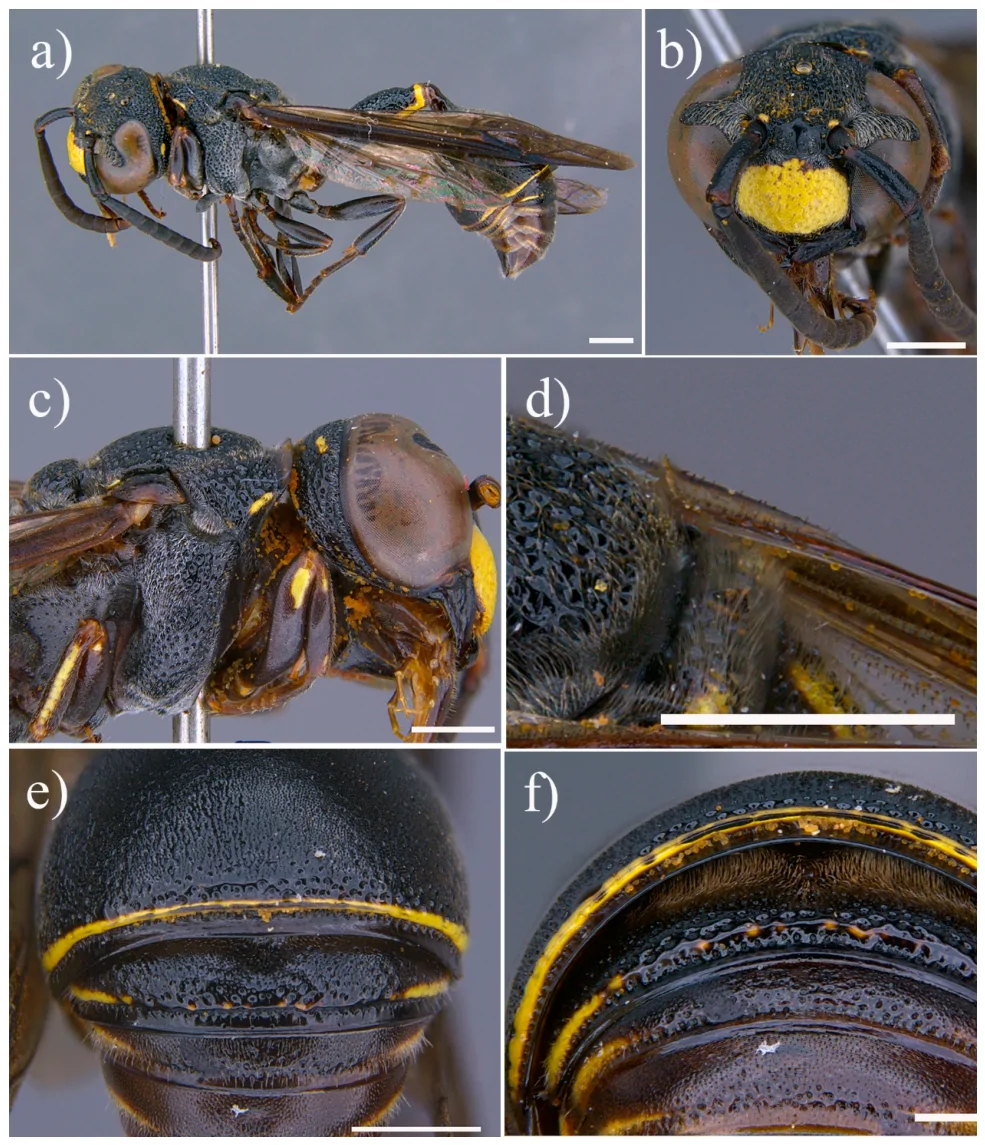
*Zethus* (*Zethittus*) *ramphoceros*. (**a**) male habitus; (**b**) clypeus and internantennal carina with elevation area; (**c**) male in lateral view; (**d**) metanotum with a weakly and robust tubercle; (**e**) T2 apical band of macropunctation and (**f**) T3 gradulus. Scale bars: 1.0 mm.

**Figure 6:**
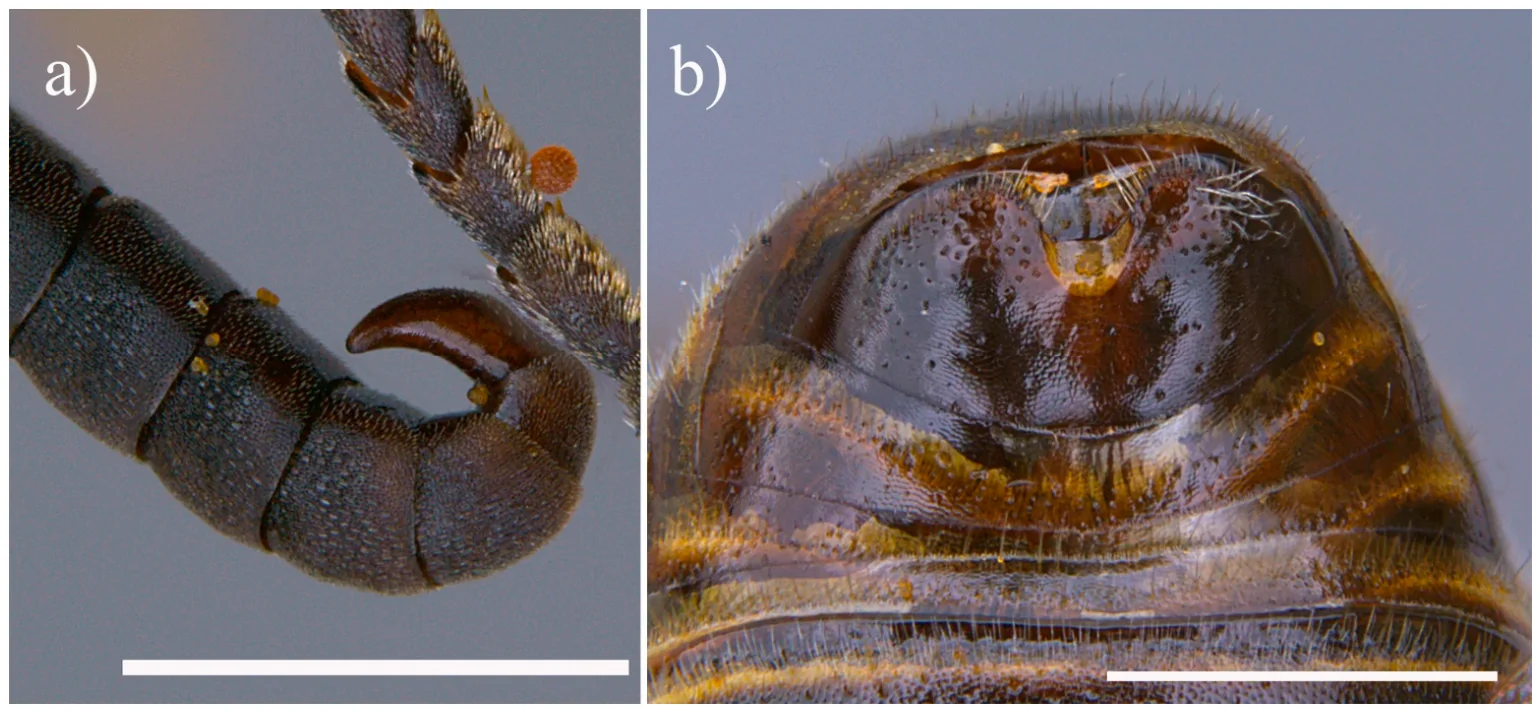
*Zethus* (*Zethittus*) *ramphoceros*. (**a**) a unique shape of the last flagellomere in its similarity. to a toucan; (**b**) S7 with a rectangular notch and a translucent lamella. Scale bars: 1.0 mm.

**Diagnosis:** Interantennal carinae developed forming a distinctive median elevation; metanotum with a weak robust and flattening tubercle and S7 with a rectangular notch (Figure 5 and Figure 6).

**Description:** MALE. *Coloration*. Black and polished with the following yellow markings: clypeus (Figure 5); antennal scape; small spots on gena; lateral bands on pronotum; parategula; broad vertical bands on propodeum; fore and mid legs with transverse yellow bands on femora and tibiae; T1 with a subapical yellow band laterally prolonged to the middle of the petiole; T2–T6 with subapical yellow bands; and S2–S6 also with subapical yellow bands. Wings ferruginous. 

*Sculpture:* The body almost with dense macropunctation, including head, gena, frons, vertex, pronotum, mesoscutum, mesoscutellum, metanotum and mesopleura; clypeus with sparse macropunctures. Interantennal carinae present and forming an elevation in the area (Figure 5). Epicnemial carinae incomplete; median region of propodeum striate and lateral region showing a sparse macropunctation. T1 densely macropunctate; T2 strongly micropunctate and some longitudinal striae with a regular apical band of macropunctures, a characteristic feature of *Zethus* (*Zethitthus*) (Figure 5). Subapical margin on T2 medially angled and lamella present.

*Structure:* Clypeus concave and weakly tridentade; pronotal lamella broad and straight without interruptions; scutellum with rounded lateral margins, lacking carinae; and metanotum with a weak and flattening median tubercle (Figure 5). Propodeal orifice and apical propodeal lamella present. Petiole highest after middle; T2 projected medially; and T3 with gradulus (Figure 5). S7 with a notch, but it is not wide, more rectangular and lamella is developed (Figure 6). Wing lengths: 5,7 mm.

*Pilosity:* In general, the pilosity is of medium length, except on the propodeum where the setae are very dense, short and silvery, having the same pilosity as the T3 gradulus.

**Figure 7:**
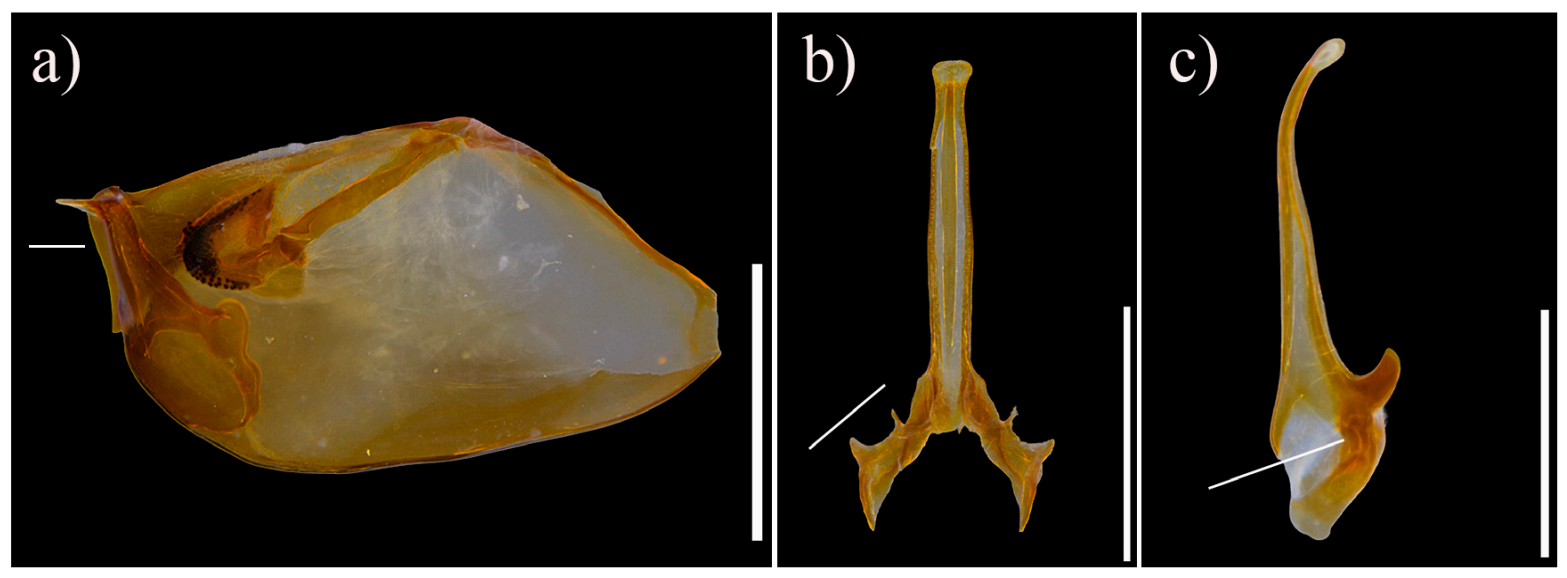
*Zethus* (*Zethittus*) *ramphocerus*. The white line shows (**a**) The wide gonocoxite, exhibit the digitus and cuspis; (**b**) Aedeagus, the apodemes are curved outward and have a triangular, pointed shape; (**c**) The developed and slender basal plate is present, with a conical and slender shape. Scale bars: 1.0 mm.

*Genitalia*: A developed, but short and slender basal plate is present, with a conical shape and most clearly visible in lateral view (Figure 7). The apodemes are curved outward and have a triangular, pointed shape. The ventral lobe is directed inward and bears a serrated portion. The gonocoxite is broad and rounded, with a bilobed apex exhibiting the same degree of symmetry on both sides. The distal lobe is elongated, extending close to the gonostylus, with a rounded apex and no visible setae. The gonostylus is short and slightly sinuous, although predominantly straight in appearance. The digitus and cuspis are similar in size. The cuspis is digitiform and associated with a large basal lobe (Figure 7).

**Etymology:** The name combines the greek words ramphos, meaning beak, and ceratus, meaning horn, recalling the antennae. This refers to the unique shape of the last flagellomere in its similarity to a toucan (genus *Ramphastos*) (Figure 6).

**Material examined:** Holotype, male, Brasil, Minas Gerais, Jaíba, 25 km sul, 09. IV. 1998, G.A.R. Melo [DZUP]. Paratype, male, Brasil, Minas Gerais, Jaíba, 25 km sul, 09. IV. 1998, G.A.R. Melo [DZUP].

**Distribution:** Brazil (Minas Gerais).

**Observation**: This species can be identified in the taxonomic key of Cruciol et al. [9] differing from the corresponding *Zethus anisitsii*. The key proposed by the authors can be modified at steps 12 and 13 to accommodate the new species. 

 

12. Metanotum with a thin median tubercle. Male with a large notch on S7…………………………………………………………………… *Zethus sinuostylus* Lopes, 2014.
  − Metanotum with a robust median tubercle (robust or weakly flattened). Male with a narrow or rectangular notch on S7……………………………….. 13.

13. Interantennal carinae developed, forming a distinctive median elevation; the shape of the last flagellomere in its similarity to a toucan; metanotum with a weak, wide and flattened tubercle; male S7 with a rectangular notch……………………………………………………………………………………… *Zethus ramphoceros* Cruciol e Lopes, sp. n.

− Interantenal carina absent, metanotum with a robust median tubercle, male S7 a narrow notch……………………………… *Zethus anisitsii* Brèthes, 1906.

 

New distributional record of *Zethus semiplanus* from Brazil.

 

New occurrence data about *Zethus semiplanus* Cruciol & Lopes, 2026, were obtained during the observation of material from entomological collection loans. Examination of specimens from Espírito Santo, Brazil, previously identified as *Zethus dubius*, revealed that they actually correspond to a female of *Zethus semiplanus*. In this context, the species previously recorded only from Misiones (Argentina), is now extended to Brazil (Viena, Espírito Santo). It is worth emphasizing that this identification was made through the examination of material deposited in an entomological collection, dating back to 1961. This finding highlights the invaluable role of Brazilian scientific collections in preserving historical biological material and enabling the identification, documentation and study of biodiversity across time. This new record represents a significant addition to the distribution of the subgenus *Zethus* (*Zethittus*) and suggests future biogeographic studies on the subgenera and the Atlantic Forest. 

## 4. Discussion

The first lineage to diverge is *Z. planinotus*, recovered as the sister group to all remaining species of the subgenus and characterized by the absence of the tubercle. Cruciol et al. [9] already highlighted the absence of the tubercle, a former traditional diagnosis for the group, but refrained from defining this state as plesiomorphic or a reversal. Our analysis indicates that the tubercle is a novelty exclusive to the species in the sister group of *Z. planinotus*, sustaining the first hypothesis of the absence being a plesiomorphy. The strict consensus of this clade reveals polytomy involving two terminals, *Z. dubius* and *Z. excavatus* and two clades. This polytomy is a clear consequence of *Z. excavatus*, a terminal that leaps between clades among the most parsimonious trees, probably due the missing data from the unknown male. 

Of the two clades in the forementioned polytomy, one is minute, with only *Z. platynotatus* and *Z. nicaraguensis* and marked by the weak tubercle. The other contains all remaining representatives, including *Z. spinosus*, the type-species of the subgenus and sister to all other terminals. While most lineages within this large assemblage are marked by few synapomorphies, one of the most derived clades, containing *Z. semiplanus*, *Z. lioneli*, *Z. anisitsii* and *Z. sinuostylus*, stands out. It is supported by four synapomorphies, all male genitalia related, of which three are unique and one is homoplastic. This is further evidence of the importance of the gonocoxite, the gonostylus and the aedeagus in helping to better understand the relationship between the species in this group.

It is important to emphasize that the taxonomic knowledge of the group remains limited due to the absence of representatives of both sexes for some species. Females remain unknown for *Z. semilunaris*, *Z. lioneli*, and *Z. ramphocerus*, whereas males have not yet been recorded for *Z. excavatus*, *Z. planinotus*, and *Z. platynotatus*. This sampling gap represents an important limitation for the interpretation of morphological variation and for species delimitation. Furthermore, males are fundamental in the systematics of the táxon, as a large proportion of the diagnostic characters used in morphological analyses are associated with the male genitalia. This structure represents an important source of taxonomic information, both due to its remarkable differentiation compared with other subgenera and species groups of *Zethus* and due to the diversity of morphological traits exhibited among species. Therefore, the acquisition and examination of additional male specimens are essential for expanding the understanding of evolutionary relationships and species boundaries within the group. Nevertheless, the phylogenetic resolution obtained herein provides a consistent framework for future investigations on morphological evolution within the group, allowing character polarization and mapping throughout the evolutionary history of the recovered lineages.

## 5. Conclusions

The phylogenetic analysis recovers the monophyly of the *Zethus spinosus* group and proposes to elevate it to a new subgenus within *Zethus*, here it is recognized as *Zethus* (*Zethittus*). The importance of male genitalia for character identification and phylogenetic inference is clearly demonstrated, highlighting their value in relationships among the species within the subgenus *Zethittus*. These characters also support the recognition of the new subgenus, as several novel structures were identified that, to date, appear exclusive to this lineage. This recent discovery of new species, and the new species record further indicate that both the new subgenus *Zethittus* and the genus *Zethus* retain substantial potential for future taxonomic and evolutionary research.

## Data Availability

All the relevant material is provided within the main text and Supplementary Material of the article. Therefore, at this time, there is no need to deposit the data in additional external databases beyond the article itself.

## Supplementary Materials

Matrix constructed for the phylogenetic analysesis is provided “tnt” format. The following supporting information can be downloaded at https://www.mdpi.com/article/10.3390/insects17090898/s1.

## Appendix A

The character circumscriptions and their coding are presented below:

### Appendix A.1. Head

1. Clypeus, apical margin: (0) straight; (1) concave; (2) convex.
2. Clypeus, lateral teeth: (0) inconspicuous; (1) conspicuous.
3. Clypeus, median tooth: (0) absent; (1) present.
4. Female, clypeus striate punctate: (0) absent; (1) present.
5. Female, clypeus microstriate: (0) absent; (1) present.
6. Labial palps, number of palpomeres: (0) 4; (1) 3; (2) 2.
7. Vertex posterior to eye: (0) not elevated; (1) elevated.
8. Elevation in the ocellar region: (0) absent; (1) present.
9. Longitudinal interantennal carina: (0) absent; (1) present.
10. Transverse interantennal carina: (0) absent; (1) present.
11. Male, antenna, F-XI dorsoventral flattening: (0) absent; (1) present.
12. Male, antenna, apex of flagellum: (0) hook-shaped; (1) coiled.
13. Hypostomal carina: (0) absent; (1) present.
14. Hypostomal carina, extent: (0) posteriorly interrupted; (1) complete.
15. Gena, margin: (0) regular; (1) sinuous.

### Appendix A.2. Mesosoma

16. Pronotum, anterior face concave: (0) absent; (1) present.
17. Pronotum, pronotal lamella, extent: (0) reduced; (1) developed.
18. Tegula, elevated outer margin, extent: (0) interrupted medially; (1) posterior half; (2) complete.
19. Pretegular carinae: (0) absent; (1) present.
20. Epicnemial carinae, dorsal portion: (0) incomplete; (1) complete.
21. Mesoscutum, parapsidal lines: (0) absent; (1) present.
22. Mesoscutum, notauli: (0) absent; (1) present.
23. Female, mesoscutum, discal punctation: (0) absent; (1) present.
24. Metanotum, median tubercle: (0) absent; (1) present.
25. Metanotum, median tubercle, prominence: (0) reduced; (1) prominent.
26. Metanotum, median tubercle, shape: (0) flattened/robust; (1) tapered/slender.
27. Fore coxae, anterior carinae: (0) absent; (1) present.
28. Mid-tibia, number of apical spurs: (0) 0; (1) 1; (2) 2.
29. Hind tibia, distribution of spines: (0) scattered; (1) arranged in a row.
30. Propodeum, apical propodeal lamella: (0) absent; (1) present.
31. Propodeum, dorsal opening: (0) absent; (1) present.

### Appendix A.3. Metasoma

32. Length of T1 petiole relative to T2: (0) equal; (1) T1 > T2; (2) T1 < T2.
33. T2, apical region with macropunctate band: (0) absent; (1) present.
34. T2, apical macropunctate band, appearance: (0) regular/homogeneous; (1) irregular/uneven.
35. T2, median apical angulation: (0) absent; (1) present.
36. T2, lamella with median apical angulation: (0) absent; (1) present.
37. S3, lamella, extent: (0) reduced; (1) developed; (2) absent.
38. S3, lamella, lamellar lobe projecting subapically beyond the sclerite: (0) absent; (1) present.
39. S3, lamella, lateral lamellar dentition: (0) absent; (1) present.
40. S3, gradulus: (0) absent; (1) present.
41. Female, T6, subapical longitudinal depression: (0) absent; (1) present.
42. Female, T6, subapical longitudinal depression: (0) shallow, without lateral tubercles; (1) deep, with lateral tubercles.
43. Male, S7, notch: (0) absent; (1) present.
44. Male, S7, translucent lamella: (0) absent; (1) present.

### Appendix A.4. Male Genitalia

45. Gonocoxite, apex: (0) straight; (1) curved.
46. Gonostylus, apex: (0) straight; (1) curved.
47. Gonostylus, curved apex: (0) simple; (1) sinuous.
48. Gonostylus, arch: (0) absent; (1) present.
49. Gonostylus, arch, thickness: (0) slender; (1) robust.
50. Aedeagus, ventral lobe fused to apodeme: (0) absent; (1) present.
51. Aedeagus, ventral lobe, orientation: (0) ventrally exposed; (1) folded inward.
52. Aedeagus, ventral lobe, margin: (0) smooth; (1) toothed; (2) fringed.
53. Apophysis, shape: (0) rounded; (1) acute.
54. Apodeme, apex: (0) flattened; (1) conical.
55. Aedeagus, basal plate: (0) reduced; (1) developed.
56. Aedeagus, basal plate, shape: (0) tapered; (1) broad.
57. Aedeagus, dorsal “hump” shape: (0) absent; (1) present.
58. Digitus, basal projection: (0) absent; (1) present.
59. Cuspis, basoventral projection: (0) absent; (1) present.
60. Cuspis, apical tuft: (0) absent; (1) present.

## References

[1] Carpenter J.M., Marques O.M. (2001). Contribuição ao Estudo dos Vespídes do Brasil (Insecta, Hymenoptera, Vespoidea, Vespidae).

[2] Hermes M.G., Somavilla A. (2015). Vespidae. Catálogo Taxonômico da Fauna do Brasil.

[3] de Saussure H. (1855). Études sur la Famille des Vespides. III. La Monographie des Masariens et un Supplément à la Monographie des Euméniens.

[4] de Saussure H. (1875). Synopsis of American Wasps: Solitary Wasps.

[5] Lopes R.B., Noll F.B. (2018). Cladistic analysis of the *Zethus* (*Zethoides*) Fox, 1899 (Hymenoptera, Vespidae, Eumeninae) species groups with insights on the current subgeneric classification of *Zethus* Fabricius, 1804. Insect Syst. Evol..

[6] Bohart R.M., Stange L.A. (1965). A Revision of the Genus Zethus Fabricius in the Western Hemisphere (Hymenoptera: Eumenidae). University of California Publications in Entomology.

[7] Soika A.G. (1979). Revisione delle specie etiopiche e malgasce della sottofamiglia Discoeliinae (Hym.). Boll. Mus. Stor. Nat. Venezia.

[8] Lopes R.B., Carpenter J.M., Noll F.B. (2021). Cladistic analysis of *Zethus* Fabricius, 1804 (Hymenoptera, Vespidae): A new subgeneric classification. J. Hymenopt. Res..

[9] Cruciol A.H., Noll F.B., Stange L.A., Lopes R.B. (2026). Five new species of the Zethus spinosus species group (Hymenoptera, Vespidae, Eumeninae, Zethus). Zootaxa.

[10] Pickett K.M., Carpenter J.M. (2010). Simultaneous analysis and the origin of eusociality in the Vespidae (Insecta: Hymenoptera). Arthropod Syst. Phylogeny.

[11] Hermes M.G., Melo G.A.R., Carpenter J.M. (2014). The higher level phylogenetic relationships of the Eumeninae (Insecta, Hymenoptera, Vepidae) with emphasis on *Eumenes sensu lato*. Cladistics.

[12] Bitsch J. (2012). Morphologie comparée des derniers segments du gastre et des genitalia mâles des Vespidae. 1. Sous-famille des Eumeninae (Hymenoptera). Bull. Soc. Entomol. Fr..

[13] Nixon K.C. (2015). WinClada.

[14] Goloboff P.A., Catalano S.A. (2016). TNT version 1.5, including a full implementation of phylogenetic morphometrics. Cladistics.

[15] Goloboff P.A. (1993). Estimating character weights during tree search. Cladistics.

[16] Goloboff P.A., Carpenter J.M., Arias J.S., Esquivel D.R.M. (2008). Weighting against homoplasy improves phylogenetic analysis of morphological data sets. Cladistics.

[17] Lopes R.B., Noll F.B. (2014). Notes on the Neotropical *Zethus* Fabricius, 1804 (Hymenoptera, Vespidae, Eumeninae) with the description of two new species from Brazil. Zootaxa.

[18] Goloboff P.A., Farris J.S., Källersjö M., Oxelmann B., Ramírez M., Szumik C. (2003). Improvements to resampling measures of group support. Cladistics.

