# Supplementary material for: Cladistics of the Zethus spinosus de Saussure Species Group: A New Subgenus of Zethus Fabricius, 1804 (Hymenoptera, Vespidae)
Source: Insects. 2026 Aug 27;17(9):898. doi: 10.3390/insects17090898 (PMC13607376; doi:10.3390/insects17090898)
Supplement: Supplementary file 1 [file insects-17-00898-s001.zip › insects-4440833-supplementary.pdf]

Supplementary Material I - The matrix character obtained in “tnt” format.

Species / Characters	00	01	02	03	04	05
Z.(I)fulva	2	0	0	0	0	1
Z.(Ztt)anisitsii	0	0	0	0	0	0
Z.(Ztt)dubius	1	1	0	0	0	0
Z.(Ztt)excavatus	1	1	0	0	0	0
Z.(Ztt)gonostylus	1	1	1	0	0	0
Z.(Ztt)nicaraguensis	1	1	0	0	0	0
Z.(Ztt)sinuostylus	0	0	0	0	0	0
Z.(Ztt)spinosus	1	1	1	0	0	0
Z.(Ztt)semiplanus	2	1	1	0	0	0
Z.(Ztt)semilunaris	2	1	1	?	?	0
Z.(Ztt)lioneli	2	1	1	?	?	0
Z.(Ztt)planinotus	0	0	0	0	0	0
Z.(Ztt)platynotatus	1	1	0	0	0	0
Z.(Ztt)ramphocerus	1	1	0	?	?	0
Z.(Z)discoelioides	1	1	0	1	0	0
Z.(Z)coeruleopennis	1	1	0	1	0	0
Z.(Z)infundibuliformis	1	1	1	1	0	0
Z.(Z)proeminens	1	1	0	0	0	0
Z.(Z)spinipes	0	1	0	1	0	0
Z.(Z)wagneri	1	0	0	0	0	0
Z.(Zts)imperfectus	0	0	0	0	1	0
Z.(Zts)mexicanus	1	0	0	0	1	0
Z.(Zts)romandinus	0	1	1	0	1	0
Z.(D)fuscus	0	0	0	0	0	0
Z.(D)smithii	0	0	0	0	0	0
Z.(Zds)aztecus	1	1	1	1	0	0
Z.(Zds)diminutus	2	1	0	0	0	0
Z.(W)cylindricus	0	0	0	1	0	0
Z.(C)pilipalpus	0	1	1	0	0	1

Species / Characters	06	07	08	09	10	11	12
Z.(I)fulva	0	0	0	1	0	0	1
Z.(Ztt)anisitsii	0	1	0	0	1	0	1
Z.(Ztt)dubius	0	0	1	0	1	0	1
Z.(Ztt)excavatus	0	0	1	0	?	0	1
Z.(Ztt)gonostylus	0	0	1	0	1	0	1
Z.(Ztt)nicaraguensis	0	0	0	0	1	0	1
Z.(Ztt)sinuostylus	0	1	0	0	1	0	1
Z.(Ztt)spinosus	0	0	1	1	1	0	1
Z.(Ztt)semiplanus	0	1	1	0	1	0	1
Z.(Ztt)semilunaris	0	1	1	0	1	0	1
Z.(Ztt)lioneli	0	1	0	1	1	0	1
Z.(Ztt)planinotus	0	0	0	0	?	0	1
Z.(Ztt)platynotatus	0	0	0	1	?	0	1
Z.(Ztt)ramphocerus	0	1	1	1	1	0	1
Z.(Z)discoelioides	1	0	0	0	1	0	1
Z.(Z)coeruleopennis	1	1	1	0	0	0	1
Z.(Z)infundibuliformis	1	0	0	0	0	0	0
Z.(Z)proeminens	1	0	0	0	0	0	1
Z.(Z)spinipes	1	0	0	0	1	0	0
Z.(Z)wagneri	1	0	0	0	?	0	0
Z.(Zts)imperfectus	0	0	0	0	0	1	1
Z.(Zts)mexicanus	1	1	0	0	0	1	1
Z.(Zts)romandinus	1	1	0	0	0	1	1
Z.(D)fuscus	0	0	1	1	0	0	1
Z.(D)smithii	0	0	1	1	0	0	1
Z.(Zds)aztecus	0	0	1	0	?	0	1
Z.(Zds)diminutus	0	0	1	1	?	0	1
Z.(W)cylindricus	0	0	1	1	1	0	1
Z.(C)pilipalpus	0	0	1	1	0	0	1

**Species / Characters**	**13**	**14**	**15**	**16**	**17**	**18**	**19**
Z.(I)fulva	0	0	0	0	1	0	1
Z.(Ztt)anisitsii	1	0	0	1	1	1	0
Z.(Ztt)dubius	1	0	0	1	1	1	0
Z.(Ztt)excavatus	1	0	0	1	1	1	0
Z.(Ztt)gonostylus	1	0	0	1	1	1	0
Z.(Ztt)nicaraguensis	1	0	0	1	1	1	0
Z.(Ztt)sinuostylus	1	0	0	1	1	1	0
Z.(Ztt)spinosus	1	0	0	1	1	1	0
Z.(Ztt)semiplanus	1	0	0	1	1	1	0
Z.(Ztt)semilunaris	1	0	1	1	1	1	0
Z.(Ztt)lioneli	1	0	0	1	1	1	0
Z.(Ztt)planinotus	1	0	0	1	1	1	0
Z.(Ztt)platynotatus	1	0	0	1	1	1	0
Z.(Ztt)ramphocerus	1	0	0	1	0	1	0
Z.(Z)discoelioides	1	0	0	1	1	0	1
Z.(Z)coeruleopennis	0	0	0	1	1	0	0
Z.(Z)infundibuliformis	-	0	1	1	-	1	0
Z.(Z)proeminens	0	0	0	0	1	0	1
Z.(Z)spinipes	-	0	0	1	1	0	1
Z.(Z)wagneri	-	0	0	0	0	1	1
Z.(Zts)imperfectus	1	1	0	1	0	1	1
Z.(Zts)mexicanus	1	0	0	1	0	1	1
Z.(Zts)romandinus	1	0	0	1	2	1	1
Z.(D)fuscus	1	0	0	1	1	1	0
Z.(D)smithii	1	0	0	1	2	1	1
Z.(Zds)aztecus	1	0	0	1	2	0	1
Z.(Zds)diminutus	1	1	0	1	2	1	1
Z.(W)cylindricus	0	1	0	1	1	1	1
Z.(C)pilipalpus	1	0	0	1	1	1	1

Species / Characters	20	21	22	23	24	25	26
Z.(I)fulva	0	0	1	0	-	-	0
Z.(Ztt)anisitsii	1	0	0	1	0	0	1
Z.(Ztt)dubius	1	0	0	1	1	1	1
Z.(Ztt)excavatus	1	0	0	1	1	1	1
Z.(Ztt)gonostylus	1	0	0	1	1	1	1
Z.(Ztt)nicaraguensis	1	0	0	1	0	0	1
Z.(Ztt)sinuostylus	1	0	1	1	0	1	1
Z.(Ztt)spinosus	1	0	0	1	1	1	1
Z.(Ztt)semiplanus	1	0	1	1	0	0	1
Z.(Ztt)semilunaris	1	0	?	1	1	0	1
Z.(Ztt)lioneli	1	0	?	1	0	0	1
Z.(Ztt)planinotus	0	0	0	0	-	-	1
Z.(Ztt)platynotatus	1	0	1	1	0	1	1
Z.(Ztt)ramphocerus	1	0	?	1	0	0	1
Z.(Z)discoelioides	1	1	1	0	-	-	0
Z.(Z)coeruleopennis	1	1	1	0	-	-	0
Z.(Z)infundibuliformis	0	1	0	0	-	-	0
Z.(Z)proeminens	1	1	0	0	-	-	0
Z.(Z)spinipes	0	1	1	0	-	-	0
Z.(Z)wagneri	1	1	1	0	-	-	0
Z.(Zts)imperfectus	0	0	0	0	-	-	1
Z.(Zts)mexicanus	1	0	0	0	-	-	1
Z.(Zts)romandinus	1	0	0	0	-	-	1
Z.(D)fuscus	1	0	1	0	-	-	0
Z.(D)smithii	1	0	1	0	-	-	0
Z.(Zds)aztecus	0	0	0	0	-	-	0
Z.(Zds)diminutus	0	0	1	0	-	-	0
Z.(W)cylindricus	1	1	1	0	-	-	0
Z.(C)pilipalpus	0	1	1	0	-	-	0

Species / Characters	27	28	29	30	31	32	33
Z.(I)fulva	2	1	1	0	1	0	-
Z.(Ztt)anisitsii	2	0	0	1	1	1	0
Z.(Ztt)dubius	2	0	0	1	1	1	0
Z.(Ztt)excavatus	2	0	0	1	1	1	0
Z.(Ztt)gonostylus	2	0	0	1	1	1	0
Z.(Ztt)nicaraguensis	2	0	0	1	1	1	1
Z.(Ztt)sinuostylus	2	0	0	1	1	1	0
Z.(Ztt)spinosus	2	0	0	1	1	1	0
Z.(Ztt)semiplanus	2	0	0	1	1	1	0
Z.(Ztt)semilunaris	2	0	0	1	1	1	0
Z.(Ztt)lioneli	2	0	0	1	1	1	1
Z.(Ztt)planinotus	2	0	0	1	1	1	0
Z.(Ztt)platynotatus	2	0	0	1	1	1	0
Z.(Ztt)ramphocerus	2	0	0	1	1	1	-
Z.(Z)discoelioides	2	0	0	1	1	0	-
Z.(Z)coeruleopennis	2	0	0	0	1	0	-
Z.(Z)infundibuliformis	2	0	0	1	1	0	-
Z.(Z)proeminens	2	0	1	0	1	0	-
Z.(Z)spinipes	1	0	0	1	1	0	-
Z.(Z)wagneri	2	0	0	0	1	0	0
Z.(Zts)imperfectus	1	0	1	1	0	1	-
Z.(Zts)mexicanus	1	0	0	0	1	0	-
Z.(Zts)romandinus	1	0	0	0	2	0	-
Z.(D)fuscus	2	1	0	1	2	0	-
Z.(D)smithii	2	1	0	1	2	0	-
Z.(Zds)aztecus	1	1	0	1	2	0	-
Z.(Zds)diminutus	1	1	1	1	1	0	1
Z.(W)cylindricus	2	1	1	1	1	1	-
Z.(C)pilipalpus	2	1	1	1	1	0	1

**Species / Characters**	**34**	**35**	**36**	**37**	**38**	**39**	**40**
Z.(I)fulva	0	0	0	0	0	0	0
Z.(Ztt)anisitsii	1	1	1	0	0	1	1
Z.(Ztt)dubius	0	0	1	0	0	1	1
Z.(Ztt)excavatus	0	0	1	0	0	1	1
Z.(Ztt)gonostylus	0	0	1	0	0	1	1
Z.(Ztt)nicaraguensis	0	0	1	0	0	1	1
Z.(Ztt)sinuostylus	1	1	1	0	0	?	1
Z.(Ztt)spinosus	0	0	1	0	0	1	1
Z.(Ztt)semiplanus	0	0	1	0	0	1	1
Z.(Ztt)semilunaris	0	0	1	0	0	1	?
Z.(Ztt)lioneli	1	1	1	0	0	1	?
Z.(Ztt)planinotus	0	0	1	0	0	?	1
Z.(Ztt)platynotatus	0	0	1	0	0	1	1
Z.(Ztt)ramphocerus	1	1	1	0	0	1	?
Z.(Z)discoelioides	0	0	1	0	0	1	0
Z.(Z)coeruleopennis	0	0	1	0	0	1	0
Z.(Z)infundibuliformis	0	0	1	0	0	1	0
Z.(Z)proeminens	0	0	1	0	0	0	0
Z.(Z)spinipes	0	0	1	0	0	1	0
Z.(Z)wagneri	0	0	1	0	0	?	0
Z.(Zts)imperfectus	0	0	1	0	0	1	0
Z.(Zts)mexicanus	0	0	0	2	0	0	0
Z.(Zts)romandinus	0	0	1	0	0	1	0
Z.(D)fuscus	0	0	1	0	0	1	0
Z.(D)smithii	0	0	1	0	0	1	0
Z.(Zds)aztecus	0	0	0	1	1	1	0
Z.(Zds)diminutus	0	0	0	1	1	1	0
Z.(W)cylindricus	0	0	1	0	0	0	0
Z.(C)pilipalpus	0	0	0	0	0	1	0

Species / Characters	41	42	43	44	45	46	47
Z.(I)fulva	-	0	0	1	0	-	0
Z.(Ztt)anisitsii	1	1	1	1	1	1	1
Z.(Ztt)dubius	0	1	1	0	1	0	1
Z.(Ztt)excavatus	0	1	1	1	1	0	?
Z.(Ztt)gonostylus	0	1	1	0	1	0	1
Z.(Ztt)nicaraguensis	0	1	1	1	1	0	1
Z.(Ztt)sinuostylus	1	1	1	1	1	1	1
Z.(Ztt)spinosus	0	1	1	0	1	0	1
Z.(Ztt)semiplanus	0	1	1	1	1	1	1
Z.(Ztt)semilunaris	?	1	1	1	1	?	?
Z.(Ztt)lioneli	?	1	1	1	1	1	1
Z.(Ztt)planinotus	0	?	?	?	?	?	?
Z.(Ztt)platynotatus	0	?	?	?	?	?	?
Z.(Ztt)ramphocerus	?	1	1	0	1	0	1
Z.(Z)discoelioides	-	0	0	1	0	-	0
Z.(Z)coeruleopennis	-	0	0	1	0	-	0
Z.(Z)infundibuliformis	-	0	0	1	0	-	0
Z.(Z)proeminens	-	0	0	1	0	-	0
Z.(Z)spinipes	-	0	0	1	0	-	0
Z.(Z)wagneri	-	0	0	?	?	-	?
Z.(Zts)imperfectus	-	0	0	1	0	-	0
Z.(Zts)mexicanus	-	0	0	1	0	-	0
Z.(Zts)romandinus	-	0	0	1	0	-	0
Z.(D)fuscus	-	0	0	1	0	-	0
Z.(D)smithii	-	0	0	1	0	-	0
Z.(Zds)aztecus	-	0	0	?	?	-	?
Z.(Zds)diminutus	-	0	0	1	0	-	0
Z.(W)cylindricus	-	0	0	0	0	-	0
Z.(C)pilipalpus	-	0	0	1	0	-	0

Species / Characters	48	49	50	51	52	53	54
Z.(I)fulva	-	0	0	?	0	1	1
Z.(Ztt)anisitsii	0	0	1	0	0	0	0
Z.(Ztt)dubius	1	1	0	0	1	1	1
Z.(Ztt)excavatus	?	?	?	?	?	?	?
Z.(Ztt)gonostylus	1	0	0	0	1	1	1
Z.(Ztt)nicaraguensis	1	1	0	0	1	0	1
Z.(Ztt)sinuostylus	0	0	1	0	0	0	0
Z.(Ztt)spinosus	1	0	0	0	1	1	0
Z.(Ztt)semiplanus	0	0	1	0	0	0	0
Z.(Ztt)semilunaris	?	?	?	?	?	?	?
Z.(Ztt)lioneli	0	0	1	0	1	0	0
Z.(Ztt)planinotus	?	?	?	?	?	?	?
Z.(Ztt)platynotatus	?	?	?	?	?	?	?
Z.(Ztt)ramphocerus	1	1	1	1	1	1	1
Z.(Z)discoelioides	-	0	0	1	0	1	1
Z.(Z)coeruleopennis	-	0	0	1	0	1	1
Z.(Z)infundibuliformis	-	0	0	0	0	1	1
Z.(Z)proeminens	-	0	0	1	1	1	1
Z.(Z)spinipes	-	0	0	1	0	1	1
Z.(Z)wagneri	?	?	?	?	?	?	?
Z.(Zts)imperfectus	-	0	0	0	0	1	1
Z.(Zts)mexicanus	-	0	0	1	0	1	1
Z.(Zts)romandinus	-	0	0	1	0	1	1
Z.(D)fuscus	-	0	0	2	0	1	1
Z.(D)smithii	-	0	0	1	0	1	1
Z.(Zds)aztecus	?	0	0	-	0	1	1
Z.(Zds)diminutus	-	0	0	?	1	1	1
Z.(W)cylindricus	-	0	0	0	0	1	1
Z.(C)pilipalpus	-	0	0	0	0	0	1

Species / Characters	55	56	57	58	59
Z.(I)fulva	0	0	0	?	?
Z.(Ztt)anisitsii	-	1	0	1	0
Z.(Ztt)dubius	1	0	0	1	0
Z.(Ztt)excavatus	?	?	?	?	?
Z.(Ztt)gonostylus	1	0	0	1	0
Z.(Ztt)nicaraguensis	1	0	0	1	0
Z.(Ztt)sinuostylus	1	1	0	1	0
Z.(Ztt)spinosus	1	0	0	1	0
Z.(Ztt)semiplanus	1	1	0	1	0
Z.(Ztt)semilunaris	?	?	?	?	?
Z.(Ztt)lioneli	1	1	0	1	0
Z.(Ztt)planinotus	?	?	?	?	?
Z.(Ztt)platynotatus	?	?	?	?	?
Z.(Ztt)ramphocerus	0	0	0	1	0
Z.(Z)discoelioides	1	0	0	0	0
Z.(Z)coeruleopennis	1	0	1	0	0
Z.(Z)infundibuliformis	1	0	1	0	0
Z.(Z)proeminens	1	0	1	0	0
Z.(Z)spinipes	1	0	0	1	0
Z.(Z)wagneri	?	?	?	?	?
Z.(Zts)imperfectus	1	0	0	1	1
Z.(Zts)mexicanus	1	0	0	1	1
Z.(Zts)romandinus	1	0	0	1	1
Z.(D)fuscus	0	0	0	0	0
Z.(D)smithii	1	0	0	0	0
Z.(Zds)aztecus	1	0	0	1	0
Z.(Zds)diminutus	1	0	0	1	0
Z.(W)cylindricus	1	0	0	1	0
Z.(C)pilipalpus	1	0	1	1	0
